# Transitions in Latent Classes of Sexual Risk Behavior Among Young Injection Drug Users Following HIV Prevention Intervention

**DOI:** 10.1007/s10461-013-0601-2

**Published:** 2013-08-22

**Authors:** Mary Ellen Mackesy-Amiti, Lawrence J. Ouellet, Lorna Finnegan, Holly Hagan, Elizabeth Golub, Mary Latka, Karla Wagner, Richard S. Garfein

**Affiliations:** 1 Division of Epidemiology and Biostatistics, School of Public Health, Community Outreach Intervention Projects , University of Illinois at Chicago, 1603 W. Taylor St., MC 923, Chicago, IL 60612 USA; 2 Department of Health Systems Science, College of Nursing, University of Illinois at Chicago, Chicago, IL USA; 3 Center for Drug Use and HIV Research, National Development and Research Institutes, New York, NY USA; 4 Bloomberg School of Public Health, Johns Hopkins University, Baltimore, MD USA; 5 The Aurum Institute, Johannesburg, South Africa; 6 Division of Global Public Health, School of Medicine, University of California, San Diego, CA USA

**Keywords:** HIV, Prevention, Injection drug use, Sexual risk behavior, Latent class analysis

## Abstract

We analyzed data from a large randomized HIV/HCV prevention intervention trial with young injection drug users (IDUs). Using categorical latent variable analysis, we identified distinct classes of sexual behavior for men and women. We conducted a latent transition analysis to test the effect of the intervention on transitions from higher to lower risk classes. Men who were in a high-risk class at baseline who received the intervention were 86 % more likely to be in a low-risk class at follow-up compared to those in the control group (*p* = 0.025). High-risk intervention participants were significantly more likely to transition to the class characterized by unprotected sex with a main partner only, while low-risk intervention participants were significantly less likely to transition to that class. No intervention effect was detected on the sexual risk behavior of women, or of men who at baseline were having unprotected sex with a main partner only.

**Electronic supplementary material:**

The online version of this article (doi:10.1007/s10461-013-0601-2) contains supplementary material, which is available to authorized users.

## Resumen

Analizamos los datos de un ensayo grade de intervención aleatorizado de prevención del VIH/VHC con jóvenes usuarios de drogas inyectables (UDI), Utilizando el análisis de variable latente categórico, se identificaron distintas clases de comportamiento sexual para hombres y mujeres. Se realizó un análisis de transición latente para probar el efecto de la intervención sobre las transiciones de alto riesgo a las clases de menor riesgo. Los hombres que estaban en una clase de alto riesgo al inicio del estudio que recibieron la intervención eran 86 % más propensos a estar en una clase de bajo riesgo durante el seguimiento en comparación con los del grupo control (*p* = 0.025). Participantes en la intervención de alto riesgo tuvieron significativamente más probabilidades de transición a la clase que se caracteriza por relaciones sexuales sin protección con sólo una pareja principal, mientras que los participantes de intervención de bajo riesgo tuvieron significativamente menos probabilidades de transición a esa clase. Ningún efecto de la intervención fue detectado en el comportamiento de riesgo sexual de las mujeres o de los hombres que al inicio del estudio estaban teniendo relaciones sexuales sin protección con una pareja principal solamente.

## Introduction

As HIV prevention efforts have achieved significant reductions in syringe-sharing among injection drug users (IDUs), attention has turned to the importance of addressing sexual transmission of HIV in this population [1–9]. High-risk behavior including exchange sex [10, 11] and anal sex [12–16] is associated with increased HIV transmission among IDUs, and is a potential bridge to non-IDU populations [17–20]. However, interventions with IDUs have often been less effective in reducing sexual risk behavior than injection risk behavior [21, 22].

The Third Collaborative Injection Drug Users Study (CIDUS-III) Drug Users Intervention Trial (DUIT), conducted from 2002 through 2005 in five cities, is the largest randomized HIV prevention intervention trial with young IDUs in the US to date. This study compared a peer education intervention (PEI) with a time-matched, attention control group receiving standard counseling and testing. The DUIT enhanced intervention demonstrated an overall greater decrease in injection-related HIV risk behavior compared to the control [23, 24]; however, it did not appear to have any greater effect on sexual risk behavior than the control.

The measures used for sexual risk behavior in that analysis were numbers of unprotected sex acts, including total number, and broken down by sex act (vaginal or anal) and partner type (main, other steady, casual/sex trade). However, sexual risk behavior is multi-dimensional, and is comprised of various combinations of behaviors (i.e., oral sex, anal sex, vaginal sex), partner types (i.e., casual, steady, exchange), and use of preventive measures (i.e., condom use). Participants exhibit different combinations of risk behaviors, and interventions may affect patterns of behavior in ways that one-dimensional measures do not capture. We conducted a secondary analysis of sexual risk behavior outcomes in the DUIT data to test the effect of the intervention on transitions from higher to lower risk classes at follow-up. To capture the multi-dimensional aspects of sexual risk behavior, we used latent class analysis to identify distinct classes of sexual risk among men and women. We then used latent transition analysis to investigate the effects of the intervention within each of these classes.

## Methods

### Study Design

We analyzed existing CIDUS-III/DUIT data collected between May 2002 and January 2004 from participants who were recruited in five US cities: Baltimore, MD; Chicago, IL; Los Angeles, CA; New York City, NY; and Seattle, WA. Details of the study objectives, design and methodology have been described elsewhere [25, 26]. Participants were eligible for the trial if they reported injecting illicit drugs in the past 6 months, intended to reside in their recruitment city for at least the next 12 months, spoke English, were between 15 and 30 years old, and tested antibody-negative for HIV and HCV at baseline (*N* = 2,062). Eligible participants who attended the post-test counseling session (*N* = 1,564) were invited to participate in the trial. Individuals who consented to participate in the trial (*N* = 854) were randomly assigned to either the PEI, or a video-discussion control group. Participants in both conditions attended six group sessions over a three-week period. All participants attended at least the first session; attendance at each of the remaining sessions was reasonably high and similar across trial arms (average 77 % for PEI, 78 % for control). Participants were compensated for time and travel after each visit, according to local guidelines—$20–40 for behavioral assessment interviews, $10–15 for each test result visit, and $20–25 for each intervention session attended (with four sites offering a $40 bonus for attending all six sessions).

PEI participants were informed that the purpose of the intervention was to train them to be peer educators who could help in the fight against AIDS and hepatitis in their communities. Talking to others about HIV and HCV prevention, in a pro-social role of peer educator, was expected to motivate behavior change in the educators [26]. In the first four sessions, participants learned what it meant to be a peer educator and were given tools appropriate to this role. The first two sessions focused on injection-related risk and the third and fourth sessions focused on sexual risk behavior. The format included videos; interactive discussions; exercises in skills building, role playing, and practice; and other factors such as offering community resources, information, and tools (e.g., condoms) at every session. In the fifth session, participants were given an opportunity to practice sharing risk-reduction information in a community setting, for example, by engaging in supervised peer outreach or staffing an information table at a community center or health fair. These experiences were followed by debriefing and feedback from the intervention facilitator in a community setting. The sixth session consisted of a group debriefing about the community-based peer education session, followed by a goal-setting activity.

The control condition consisted of watching videos followed by facilitated discussion for an equivalent amount of time as the PEI sessions. Videos addressing social and health issues were chosen to be of interest to the target population, yet devoid of specific HIV/HCV risk-reduction content.

At baseline and follow-up visits, participants completed a behavioral assessment using audio computer-assisted self-interview (ACASI) technology to minimize socially desirable responding. Retention rates for the three- and six-month follow-up visits were 64 and 76 %, respectively, with 83 % of the sample (*N* = 712) completing at least one follow-up interview. The most common reasons for loss to follow-up were entering drug treatment (32 %), moving out of the area (27 %), and incarceration (15 %). It was previously reported that loss to follow-up was unrelated to trial arm assignment or targeted risk behaviors [25]. Institutional review boards at the CDC and all collaborating institutions approved the study protocol, and all individuals provided written, informed consent to participate in the study.

### Measures

#### Sociodemographic Measures

Respondents provided information on sociodemographic characteristics, including sex, age, race/ethnicity, homelessness, incarceration, and sources of income (legal and illegal).

#### Sexual Risk Behavior

Participants were asked about their sexual activities in the previous three months, including numbers of steady and casual partners, exchanging sex for money or drugs, condom use during vaginal, anal, and oral sex with steady and casual partners, and condom use during exchange sex (see Table 1).

**Table 1:** Candidate measures and final selected measures for latent class analysis

Initial candidate measures	Selected measures	
	Male	Female
Number of steady female sex partners	Number of female sex partners (none, 1, >1)	NA
Number of casual female sex partners		NA
Number of steady male sex partners	Any male sex partner	Multiple male sex partners (vs. 0 or 1)
Number of casual male sex partners		Number of casual sex partners (none, 1, >1)
Gave money or drugs in exchange for sex	Not included	NA
Received money or drugs in exchange for sex	Included as is	Included as is
Condom use with sex trade partners (5 point ordinal scale)	Any unprotected trade sex	Any unprotected trade sex
Any unprotected vaginal sex with main partners	Included as is	Included as is
Any unprotected heterosexual anal sex with main partner	Included as is	Included as is
Any unprotected vaginal sex with other steady partners	Any unprotected vaginal sex with non-main partner	Any unprotected vaginal or anal sex with non-main partner
Any unprotected vaginal sex with casual partners		
Any unprotected heterosexual anal sex with other steady partners	Any unprotected heterosexual anal sex with non-main partner	
Any unprotected heterosexual anal sex with casual partners		
Any unprotected anal sex with main male partner (MSM)	Included as is	NA
Any unprotected anal sex with other steady male partners (MSM)	Any unprotected anal sex with non-main partner (MSM)	NA
Any unprotected anal sex with casual male partners (MSM)		NA

#### Analysis

Beginning with a set of 16 variables for men, and 10 variables for women, we conducted exploratory latent class analyses with the baseline data of participants who were invited to participate in the trial (unpublished data). We explored models with two to seven classes using all measures, and systematically eliminated variables and levels of variables that did not distinguish between classes, tested categorical variables derived from count measures, and combined variables that were highly collinear. Table 1 shows the initial candidate measures, and the final selected measures for men and women. Out of 16 candidate measures of male sexual risk behavior, we selected 10 for inclusion, and out of 10 candidate measures of female sexual risk behavior, we selected 7 for inclusion. The initial analyses indicated that for both men and women we could expect to extract at least three and not more than six classes.

Consistent with previous analyses of these data [23], the main analysis used data from the 712 participants who completed at least one follow-up interview. We conducted latent class analyses of sexual risk behaviors separately for men and women using Mplus version 6.1 [27]. We fit latent class models with three to six classes at each time point, and computed the Vuong-Lo-Mendell-Rubin (VLMR) [28] and bootstrap likelihood ratio tests (BLRT) [29] and compared the Bayesian Information Criterion (BIC) [30] to decide on the number of classes that best fit the data [31]. We then conducted the latent transition analyses (LTA) using baseline and 6-month follow-up data. While we did examine the class structure in the 3-month follow-up data, we did not include the 3-month data in the LTA model. To assess the consistency of class structure over time, models with measurement thresholds constrained to be equal over time were compared with models allowing thresholds to vary, using the Satorra-Bentler Chi square difference test based on log-likelihood values and scaling correction factors obtained with the MLR estimator in Mplus [32]; see http://www.statmodel.com/chidiff.shtml. Finally, we added the intervention effect to the model as a known class variable, and compared a model with equal transition slopes across intervention arm (i.e. group main effect only) to a model with unequal transition slopes; that is, we tested the moderating effect of intervention arm on the multinomial regression of follow-up class on baseline class (see Figure S1, in Supplementary Material).

The probabilities of risk class membership at follow-up were further analyzed in Stata 12 using generalized linear models (glm procedure), specifying a binomial distribution and logit link function, and robust (sandwich) variance estimator. Predictors included intervention arm, most likely class at baseline, and their interaction. Contrasts were computed for the effect of intervention arm within risk class.

## Results

### Sample Demographics

The sample of DUIT participants who completed at least one follow-up interview (*N* = 712) was 65 % male, 63 % non-Hispanic White, 17 % Hispanic, and 20 % other race/ethnicity. The mean age was 24, ranging from 15 to 30 years. Forty percent reported being homeless at some point in six months before baseline and 17 % reported spending some time in jail during that period. Sexual behaviors in the past six months at baseline are shown in Table 2.

**Table 2:** Baseline sexual behavior past 6 months

Measure	Male	Female
	*N* = 466 (%)	*N* = 246 (%)
Steady female partners		
One	51.2	11.1
More than one	25.4	3.7
Casual female partners		
One	26.1	5.4
More than one	26.3	3.3
Steady male partners		
One	4.0	66.8
More than one	3.6	19.3
Casual male partners		
One	2.7	17.4
More than one	4.9	25.6
Gave money or drugs for sex	6.7	3.3
Received money or drugs for sex	10.5	22.8
Unprotected sex with trade partners	7.5	6.5
Unprotected vaginal sex, main partner	60.7	73.2
Unprotected heterosexual anal sex, main partner	20.2	20.7
Unprotected vaginal sex, other steady partners	13.7	11.4
Unprotected vaginal sex, casual partners	27.3	19.1
Unprotected heterosexual anal sex, other steady partners	5.8	0.4
Unprotected heterosexual anal sex, casual partners	10.5	6.5
Unprotected anal sex, main male partner (MSM)	1.7	NA
Unprotected anal sex, other steady male partners (MSM)	0.4	NA
Unprotected anal sex, casual male partners (MSM)	2.4	NA

### Male Sexual Risk Behavior

In the latent class analyses of baseline data, the BIC pointed to a model with five classes, and the BLRT indicated significant improvement in fit compared to the four class model (BLRT(12) = 79.91, *p* < 0.0001). The five classes included (1) a low risk group comprised of men who reported no unprotected sex (includes not sexually active) (28 %); (2) men who had unprotected sex with a main female partner only (30 %); (3) men who had unprotected sex with main and other female partners (29 %); (4) a high-risk group including men who have sex with men and women, and men who engaged in sex trade (6 %); and (5) men who have sex with men or engage in sex trade, and have low probability of unprotected sex with women (7 %).

We then estimated a latent transition model with five classes. Although the class structure was invariant over time, the thresholds for the fifth class were changed slightly in the LTA model compared to the LCA model, now indicating no unprotected sex with women in this class. The class size shrunk from 7 to 4 %, and few men transitioned into or out of this class. Consequently, since the estimates for this class would have low reliability, we decided to exclude men who had sex with men only (*N* = 13) from the sample, and re-estimated the latent class models. The BIC and the VLMR likelihood ratio tests (see Table S1 in Supplementary Material) indicated that a 4-class solution fit best at each time point. We proceeded to estimate a latent transition model with four classes, and tested for non-invariance of measurement thresholds over time. The Satorra-Bentler LRT was non-significant (TRd(11) = 11.97, *p* = 0.37), indicating that the invariant model was adequate, i.e. that the class structure did not vary significantly between baseline and 6-month follow-up. Tables S2 and S3 in the Supplementary Material present subject characteristics associated with latent classes at baseline, and item probabilities associated with the LTA model.

#### Intervention Effects

We then added intervention arm as a known class variable, and tested the effect of the intervention by comparing a model with equal transition slopes across intervention arm to a model with unequal transition slopes (i.e., with time by arm interaction). The likelihood-ratio test was significant (TRd(9) = 17.59, *p* = 0.04), indicating that the intervention effect varied across classes. This model had an entropy value of 0.891, indicating good classification quality. Based on the posterior probabilities, the prevalence of the “low-risk” class increased from 28 % at baseline to 47 % at follow-up, while the prevalence of the “multiple female partner” class decreased from 32 to 20 %. The “main only” class prevalence was 29 % at baseline, and 24 % at follow-up, while the “high-risk” class comprised 11 % of the sample at baseline, and 8 % at follow-up. The transition probabilities from this model are shown in Table 3. The diagonal values include participants who remained in the same class at both time points. For example, the probability of a low-risk participant remaining in the low-risk class was 77 % in the control arm and 90 % in the PEI arm. The off-diagonal values represent transitions across classes. For example, in the control arm, the probability of a high-risk participant transitioning to the low-risk class was 32 %, and in the PEI arm the probability was 31 %.

**Table 3:** Unadjusted posterior probabilities of class membership at follow-up by baseline class and intervention arm, Men (*N* = 453)

Baseline class^a^	Control				PEI				*N*
	Low-risk (%)	Main only (%)	Mult fem (%)	High-risk (%)	Low-risk (%)	Main only (%)	Mult fem (%)	High-risk (%)	
Low-risk	77	12	8	3	90	0	10	0	133
Main only	29	53	13	5	32	49	18	1	134
Multiple female	32	15	47	5	39	25	31	5	137
High-risk	32	9	14	45	31	24	0	45	49

^a^Most likely class based on posterior probabilities

The results of the generalized linear model analyses on the posterior probabilities of the outcome classes are shown in Table 4. The intervention arm by baseline risk class interaction effect was significant in three of the four models. In the analysis of the low-risk class probabilities, the overall interaction effect was non-significant; there was a trend for baseline low-risk class (chi^2^ = 3.06, *p* = 0.08), such that PEI participants were more likely to remain in this class (88 %) compared to “low-risk” participants in the control condition (77 %).

**Table 4:** Predicted probabilities and contrasts, generalized linear model analysis, adjusted for age, race/ethnicity, and city (men)

Outcome	Baseline class^a^	Pred. Prob.				95 % Conf. Int.		chi^2^	*p*
		Control	PEI	OR	Std Err	LL	UL		
Low risk									
Low risk		0.77	0.88	2.12	0.91	0.91	4.94	3.06	0.080
Main only		0.28	0.32	1.23	0.42	0.63	2.42	0.37	0.544
Mult female		0.32	0.35	1.14	0.39	0.58	2.24	0.15	0.695
High risk		0.35	0.37	1.09	0.63	0.36	3.37	0.02	0.876
Joint (df = 4)								3.58	0.467
Main only									
Low risk		0.10	0.01	0.10	0.06	0.03	0.31	14.98	0.000
Main only		0.57	0.49	0.70	0.23	0.37	1.32	1.21	0.272
Mult female		0.14	0.26	2.16	0.84	1.01	4.61	3.92	0.048
High risk		0.08	0.24	3.58	2.56	0.88	14.54	3.18	0.074
Joint (df = 4)								23.23	0.0001
Multi female									
Low risk		0.08	0.10	1.39	0.73	0.50	3.87	0.4	0.529
Main only		0.11	0.17	1.59	0.68	0.69	3.67	1.16	0.281
Mult female		0.49	0.31	0.46	0.15	0.25	0.87	5.8	0.016
High risk		0.16	0.02	0.09	0.06	0.02	0.35	11.76	0.001
Joint (df = 4)								19.11	0.001
High risk									
Low risk		0.05	0.00	0.01	0.01	0.00	0.08	23.66	0.000
Main only		0.04	0.02	0.51	0.43	0.10	2.67	0.63	0.429
Mult female		0.05	0.08	1.63	0.97	0.51	5.24	0.68	0.409
High risk		0.34	0.31	0.86	0.57	0.23	3.17	0.05	0.821
Joint (df = 4)								25.52	0.000

^a^Most likely class based on posterior probabilities

In the analysis of the “main only” class probabilities for men, baseline “low risk” participants in the PEI arm were significantly less likely to transition to this class than those in the control group (1 vs. 10 %, OR = 0.10, 95 % CI 0.03–0.31), and PEI participants in the “multiple female partners” risk group were significantly more likely to transition to the main only class (26 vs. 14 %, OR = 2.16, 95 % CI 1.01–4.61). There was also a trend for the “high-risk” class, with 24 % of PEI participants making this transition compared to 8 % of control participants (OR = 3.58, 95 % CI 0.88–14.54).

The analysis of the “multiple female partners” class found that PEI participants in the two higher risk classes had reduced odds of this outcome (“high risk” 2 % vs. 6 %, OR = 0.09, 95 % CI 0.02–0.35); “multiple female partners” (31 vs. 49 %, OR = 0.46, 95 % CI 0.25–0.87). For the “high risk” outcome class, “low risk” PEI participants were less likely to transition to this class (0.1 vs. 5 %, OR = 0.01, 95 % CI 0.003–0.08).

To summarize the effect of the intervention on the higher risk classes, we collapsed these two classes into one group, summed the probabilities for the two lower risk outcomes, and conducted a generalized linear model analysis of this total. Male PEI participants in the higher risk classes combined were significantly more likely (*p* = 0.025) than those in the control group to transition to either the “low risk” or “main only” class (OR = 1.86, 95 % CI 1.08–3.21).

### Female Sexual Risk Behavior

The BIC pointed to the 3-class model as the best-fitting model for both baseline and follow-up data (see Table S1 in Supplementary Material). The VLMR LRT also indicated a 3-class model at baseline, but suggested a 4-class model at 6-month follow-up. The 3-class model at both time-points identified (1) a low-risk class comprised of women who were not sexually active or had only one partner, and had either no unprotected sex or unprotected sex with a main partner only, (2) women who had more than one partner, and did not engage in trade sex, and (3) a high-risk class of women who engaged in trade sex. We proceeded to fit the latent transition model with three classes. Although there was similarity of the classes over time, there was also noticeable variability in the thresholds of several indicators. However, the likelihood ratio test for an invariant 3-class model compared to a non-invariant 3-class model indicated that the invariant model had adequate fit (TRd (24) = 23.57, *p* = 0.49). Tables S2 and S4 in the Supplementary Material present subject characteristics associated with latent classes at baseline, and item probabilities associated with the LTA model.

#### Intervention Effects

Again, we added intervention arm as a known class, and tested the effect of the intervention by comparing a model with equal transition slopes across intervention arm to a model with unequal transition slopes. The likelihood-ratio test was not significant (TRd(4) = 1.67, *p* = 0.80), indicating that the intervention effect did not vary across classes. Overall, the prevalence of the “low-risk” class based on posterior probabilities increased from 50 % at baseline to 56 % at follow-up, while the “high-risk” class decreased from 21 to 13 %. The prevalence of the “multiple partners” class remained steady at 30 % baseline and 31 % at follow-up. The transition probabilities from this model are shown in Table 5. The generalized linear model analysis of outcome probabilities also found no significant differences between intervention arms for women.

**Table 5:** Unadjusted posterior probabilities of class membership at follow-up by baseline class and intervention arm, women (*N* = 246)

Baseline class^a^	Control			PEI			*N*
	Low risk (%)	Multiple partners (%)	Trade sex (%)	Low risk (%)	Multiple partners (%)	Trade sex (%)	
Low risk	67	29	4	70	27	3	122
Multiple partners	49	31	20	43	46	11	73
Trade sex	40	31	29	41	26	33	51

^a^Most likely class based on posterior probabilities

## Discussion

The results of the latent transition analysis suggest that the DUIT PEI had an effect on the sexual risk behavior of young male IDUs other than those who were in a monogamous relationship or who used condoms outside of their main relationship. Among men in this “main only” class, about 30 % transitioned to the “low risk” class at follow-up regardless of intervention arm. Men in the PEI condition who were engaging in unprotected sex with multiple partners and other risky sexual behavior at baseline were more likely than those in the control group at follow-up to have transitioned to the “main only” class—apparently reducing their sexual risk behavior by restricting unprotected sexual activity to one main partner. At the same time, men in the PEI condition who were not engaging in unprotected sex at baseline were less likely than those in the control group at follow-up to have transitioned to the “main only” class, apparently being more likely to use condoms in a new relationship, or to continue to use condoms with their main partner. In a similar study, Latkin et al. [33] found that in a network-oriented HIV prevention intervention based on social identity theory and peer outreach, experimental compared with control group participants were more likely to report increased condom use with casual sex partners, but not with main partners.

The absence of an intervention effect on sexual risk behavior among women may reflect the lack of gender-specific content in this program. Comprehensive reviews of the effects of HIV prevention and intervention programs have found that women benefit from programs that are specifically directed toward women, and that include a focus on relationship and negotiation skills [34–37]. Research has demonstrated the importance of addressing issues of gender norms, relationship power, sexual coercion, and negotiation of safer sex for reducing HIV risk behavior among women [38–40]. While the intervention was designed to be equally relevant to women and men, and included exercises to help women negotiate condom use with male partners, issues of relationship power and intimate partner violence (not dealt with directly) could have made it more difficult for women in the study to adopt new behaviors.

While HIV prevention interventions with IDUs have shown success in reducing injection-related HIV risk behavior, research into their effectiveness in limiting sexual transmission has been less promising. The bulk of existing research on intervention effectiveness has used analysis techniques that treat the sample as a homogeneous group, and assess behavioral outcomes with one-dimensional measures (e.g. number of unprotected sex acts); even when the measures are specific (e.g., number of unprotected sex acts with casual partners), they are assessed one at a time. However, sexual risk behavior is multi-dimensional, and is comprised of various combinations of behaviors (i.e., oral sex, anal sex, vaginal sex), partner types (i.e., casual, steady, exchange), and use of preventive measures (i.e., condom use). Participants exhibit different combinations of risk behaviors, and interventions may affect patterns of behavior in ways that one-dimensional measures do not capture. For example, in this study, less than half of the men reported unprotected sex with casual partners at baseline. When we consider this, it is not surprising that the initial analysis [23] did not find a significant intervention effect. A more nuanced analysis strategy is needed to assess changes on multiple dimensions. In this analysis we used latent class analysis to identify classes of sexual risk behavior, and then investigated the effect of the PEI intervention on the probability that young IDUs transitioned in and out of these classes. This type of analysis is well-suited for capturing change in complex multi-dimensional behavior.

### Limitations

Seventeen percent of the DUIT sample was lost to follow-up. Post-hoc analyses indicated that these participants were somewhat more likely to report lower risk sexual behavior at baseline compared to those who completed a follow-up interview. However, as reported previously [23], these participants were distributed equally across trial arm.

The smaller sample size of women, as well as the smaller proportion of non-sexually active women, may have resulted in a less satisfactory solution. Fewer than 6 % of women in the DUIT sample reported no sexual activity at baseline, compared to 18.5 % of men. At the 6-month follow-up, 15.8 % of women and 32.6 % of men reported no sexual activity. Women who did not have sex were classified together with women who had unprotected sex with a main partner only, while men who did not have sex were classified together with men who always used condoms.

## Conclusions

This supplemental analysis of data from the DUIT study revealed that the PEI was at least partially effective in reducing sexual risk behavior among men, in contrast to the original analysis that found no effect. The PEI had an effect on men’s sexual behavior, reducing the likelihood of unprotected sex with a main partner among men who did not engage in unprotected sex at baseline, and reducing the likelihood of unprotected sex with non-main partners among men who engaged in risky sexual behavior at baseline. The absence of an effect among women participants highlights the need for additional activities to impact sexual risk among women. While mixture modeling should not replace univariate outcome analyses, using latent classes to model the multi-dimensional aspects of sexual risk behavior may capture changes in sexual risk behavior that would otherwise be undetected.

## Electronic supplementary material

Below is the link to the electronic supplementary material.

Supplementary material 1 (DOCX 57 kb)
