# Supplementary material for: Transitions in Latent Classes of Sexual Risk Behavior Among Young Injection Drug Users Following HIV Prevention Intervention
Source: AIDS Behav. 2013 Aug 22;18(3):464–72. doi: 10.1007/s10461-013-0601-2 (PMC3932146; doi:10.1007/s10461-013-0601-2)
Supplement: Supplementary file 1 — Supplementary material 1 (DOCX 57 kb) [file 10461_2013_601_MOESM1_ESM.docx]

**Transitions in Latent Classes of Sexual Risk Behavior Among Young Injection Drug Users Following HIV Prevention Intervention**

**SUPPLEMENTARY MATERIAL**

**Figure S1. Latent transition model for two time points with a binary covariate influencing the latent transition probabilities** (1)

The LTA model estimated is of the form shown in Figure 1. A key feature of this model is that the binary known class variable (cg) moderates the regression of c2 on c1. Transition probabilities are conditional probabilities for c2 categories given c1 categories, where c1 and c2 refer to latent class variables at two different time points. The transition probabilities are obtained from multinomial regression of c2 on c1 (2). See Mplus technical appendix for details (3).

| **Table S1. Goodness of fit and classification quality for 2- to 5-class LCA models** | | | | | | | |
| --- | --- | --- | --- | --- | --- | --- | --- |
|  | **Men** | | | | | | |
| **Classes** | **LL** | **BIC** | **adj. BIC** | **Entropy** | **-2* LLdiff^a^** | **BLRT PValue** | **VLMR Pvalue** |
| *Baseline* |  |  |  |  |  |  |  |
| 2 | -1534.85 | 3210.22 | 3137.22 | 0.83 | 471.73 | 0.0000 | 0.0000 |
| 3 | -1434.07 | 3081.96 | 2970.88 | 0.89 | 201.57 | 0.0000 | 0.0000 |
| 4 | -1357.30 | 3001.74 | 2852.58 | 0.90 | 153.53 | 0.0000 | 0.0000 |
| 5 | -1337.84 | 3036.12 | 2848.88 | 0.89 | 38.93 | 0.0000 | 0.1530 |
| *3-months* |  |  |  |  |  |  |  |
| 2 | -1037.33 | 2209.07 | 2136.11 | 0.87 | 345.68 | 0.0000 | 0.0000 |
| 3 | -956.76 | 2118.03 | 2007.01 | 0.89 | 161.16 | 0.0000 | 0.0000 |
| 4 | -903.43 | 2081.50 | 1932.40 | 0.92 | 106.66 | 0.0000 | 0.0001 |
| 5 | -888.20 | 2121.17 | 1934.01 | 0.92 | 30.46 | 0.0000 | 0.0837 |
| *6-months* |  |  |  |  |  |  |  |
| 2 | -1236.89 | 2612.03 | 2539.05 | 0.89 | 495.39 | 0.0000 | 0.0000 |
| 3 | -1147.38 | 2505.16 | 2394.10 | 0.85 | 179.01 | 0.0000 | 0.0000 |
| 4 | -1100.78 | 2484.08 | 2334.95 | 0.87 | 93.21 | 0.0000 | 0.0036 |
| 5 | -1077.85 | 2510.36 | 2323.14 | 0.90 | 45.86 | 0.0000 | 0.1935 |
|  |  |  |  |  |  |  |  |
|  | **Women** | | | | | | |
|  | **LL** | **BIC** | **adj. BIC** | **Entropy** | **-2* LLdiff^a^** | **BLRT PValue** | **VLMR Pvalue** |
| *Baseline* |  |  |  |  |  |  |  |
| 2 | -777.40 | 1648.40 | 1594.51 | 0.97 | 418.33 | 0.0000 | 0.0000 |
| 3 | -746.99 | 1637.12 | 1554.70 | 0.98 | 60.83 | 0.0000 | 0.0000 |
| 4 | -734.14 | 1660.96 | 1550.01 | 0.97 | 25.70 | 0.0000 | 0.0665 |
| 5 | -725.28 | 1692.79 | 1553.31 | 0.94 | 17.72 | 0.0300 | 0.0198 |
| *3-months* |  |  |  |  |  |  |  |
| 2 | -528.68 | 1146.91 | 1093.05 | 0.96 | 290.18 | 0.0000 | 0.0000 |
| 3 | -506.01 | 1148.99 | 1066.63 | 0.97 | 45.33 | 0.0000 | 0.0024 |
| 4 | -493.26 | 1170.90 | 1060.03 | 0.97 | 25.50 | 0.0000 | 0.0178 |
| 5 | -483.17 | 1198.12 | 1058.74 | 0.95 | 20.19 | 0.0000 | 0.0123 |
| *6-months* |  |  |  |  |  |  |  |
| 2 | -632.84 | 1357.44 | 1303.57 | 0.97 | 339.56 | 0.0000 | 0.0000 |
| 3 | -606.98 | 1354.31 | 1271.91 | 0.96 | 51.72 | 0.0000 | 0.0001 |
| 4 | -595.63 | 1380.19 | 1269.27 | 0.98 | 22.70 | 0.0000 | 0.0005 |
| 5 | -587.51 | 1412.54 | 1273.10 | 0.96 | 16.23 | 0.1364 | 0.0745 |
| ^a^ Compared to a model with one fewer classes. | | | | | | | |
| BLRT: Bootstrap likelihood ratio test | | | | | | | |
| VLMR: Vuong-Lo-Mendell-Rubin likelihood ratio test | | | | | | | |

| **Table S2. Characteristics of Baseline Latent Classes of Sexual Risk Behavior** | | | | | | | | | |
| --- | --- | --- | --- | --- | --- | --- | --- | --- | --- |
|  | Men (N=453) | | | | | Women (M=246) | | | |
|  | High risk | Multi-female | Main only | Low risk | Pvalue^a^ | Trade | Multi-partner | Low risk | Pvalue^a^ |
| Age | 23.8 | 23.8 | 23.8 | 24.4 |  | 24.2 | 22.7 | 23.2 |  |
| Hispanic | 34% | 21% | 24% | 11% | ** | 12% | 16% | 16% |  |
| Black | 11% | 10% | 10% | 5% |  | 14% | 18% | 10% |  |
| White | 49% | 64% | 63% | 80% | *** | 66% | 60% | 68% |  |
| Homeless past 6 months | 59% | 38% | 37% | 37% | † | 56% | 39% | 35% | * |
| Drink > 3 days / week | 33% | 19% | 16% | 16% |  | 20% | 20% | 10% | † |
| Binge alcohol weekly | 60% | 48% | 42% | 33% | ** | 40% | 30% | 19% | * |
| Main female partner injects | 42% | 40% | 48% | 14% | *** | 18% | 10% | 7% |  |
| Main male partner injects | 4% | 1% | 0% | 0% |  | 54% | 62% | 63% |  |
| Number of sex partners | 9.2 | 8.5 | 2.1 | 1.1 | *** | 25.7 | 6.4 | 1.1 | ** |
| Steady male partners | 0.8 | 0.0 | 0.0 | 0.0 | ** | 12.2 | 3.0 | 0.8 |  |
| Steady female partners | 3.3 | 4.3 | 1.2 | 0.6 | *** | 0.3 | 0.2 | 0.2 |  |
| Casual male partners | 1.8 | 0.0 | 0.1 | 0.0 | * | 12.8 | 3.1 | 0.0 | * |
| Casual female partners | 4.0 | 3.9 | 0.7 | 0.6 | *** | 0.3 | 0.2 | 0.1 |  |
| ^a^ Test of equality of means across latent classes using pseudo-class-based multiple imputations (4). | | | | | | | | | |
| † p < .10 * p < .05 ** p < .01 *** p < .001 | | | | | | | | | |

| **Table S3. Probabilities of items in 4-class LTA model,**  **Men (N=453)** | | | | |
| --- | --- | --- | --- | --- |
|  | Latent Class Pattern | | | |
|  | High risk | Multi-female | Main only | Low risk |
| Baseline % | 12% | 31% | 30% | 28% |
| Any male partners |  |  |  |  |
| No | 0.504 | 1 | 0.983 | 0.994 |
| Yes | 0.496 | 0 | 0.017 | 0.006 |
| Female partners |  |  |  |  |
| None | 0 | 0 | 0 | 0.668 |
| One | 0.074 | 0 | 0.768 | 0.097 |
| More than one | 0.926 | 1 | 0.232 | 0.236 |
| Trade sex |  |  |  |  |
| No | 0.432 | 0.996 | 0.984 | 0.973 |
| Yes | 0.568 | 0.004 | 0.016 | 0.027 |
| Unprotected trade sex | |  |  |  |
| No | 0.509 | 1 | 1 | 0.987 |
| Yes | 0.491 | 0 | 0 | 0.013 |
| Unprot. vag. sex, main partner | |  |  |  |
| No | 0.267 | 0.046 | 0.055 | 1 |
| Yes | 0.733 | 0.954 | 0.945 | 0 |
| Unprot. anal sex, main female | |  |  |  |
| No | 0.523 | 0.663 | 0.77 | 1 |
| Yes | 0.477 | 0.337 | 0.23 | 0 |
| Unprot. anal sex, main male | |  |  |  |
| No | 0.82 | 1 | 1 | 1 |
| Yes | 0.18 | 0 | 0 | 0 |
| Unprot. vag sex, other partner | |  |  |  |
| No | 0.397 | 0.122 | 1 | 0.982 |
| Yes | 0.603 | 0.878 | 0 | 0.018 |
| Unprot. anal sex, other female | |  |  |  |
| No | 0.533 | 0.754 | 1 | 1 |
| Yes | 0.467 | 0.246 | 0 | 0 |
| Unprot. anal sex, other male | |  |  |  |
| No | 0.843 | 1 | 1 | 1 |
| Yes | 0.157 | 0 | 0 | 0 |

| **Table S4. Probabilities of items in 3-class LTA model, Women (N=246)** | | | |
| --- | --- | --- | --- |
|  | Latent Class Pattern | | |
|  | Trade sex | Multi-partner | Low risk |
| Baseline % | 21% | 30% | 50% |
| Trade sex |  |  |  |
| No | 0 | 1 | 0.968 |
| Yes | 1 | 0 | 0.032 |
| Unprotected trade sex | |  |  |
| No | 0.658 | 1 | 1 |
| Yes | 0.342 | 0 | 0 |
| Casual male partners |  |  |  |
| None | 0.133 | 0.144 | 0.992 |
| One | 0.144 | 0.554 | 0.008 |
| More than one | 0.722 | 0.302 | 0 |
| Multiple sex partners | |  |  |
| No | 0.028 | 0.008 | 1 |
| Yes | 0.972 | 0.992 | 0 |
| Unprot. vag. sex, main partner | |  |  |
| No | 0.295 | 0.166 | 0.336 |
| Yes | 0.705 | 0.834 | 0.664 |
| Unprot anal sex., main partner | |  |  |
| No | 0.756 | 0.851 | 0.842 |
| Yes | 0.244 | 0.149 | 0.158 |
| Unprot. sex, other partner | |  |  |
| No | 0.536 | 0.41 | 1 |
| Yes | 0.464 | 0.59 | 0 |

**References**

1. Muthén LK, Muthén BO. Mplus User’s Guide. Seventh Edition. Los Angeles, CA: Muthén & Muthén; 1998-2012.

2. Muthén B, Asparouhov T. LTA in Mplus: Transition probabilities influenced by covariates. Mplus Web Notes: No 13. Los Angeles, CA: Muthén & Muthén; July 27, 2011 2011. https://www.statmodel.com/examples/LTAwebnote.pdf.

3. Asparouhov T, Muthén B. C on C and X. Technical appendix. Los Angeles, CA: Muthén & Muthén; July 26, 2011 2011. http://www.statmodel.com/download/ConC1.pdf.

4. Asparouhov T. Wald test of mean equality for potential latent class predictors in mixture modeling. Technical appendix. Los Angeles, CA: Muthén & Muthén; 2007. http://www.statmodel.com/download/MeanTest1.pdf.
